# Prevalence and Trends of Opioid Use in Patients With Depression in the United States

**DOI:** 10.7759/cureus.15309

**Published:** 2021-05-28

**Authors:** Terence Tumenta, Derek F Ugwendum, Muchi Ditah Chobufo, Etaluka Blanche Mungu, Irina Kogan, Tolulope Olupona

**Affiliations:** 1 Psychiatry, Interfaith Medical Center, Brooklyn, USA; 2 Public Health (Alumni), George Washington University, Washington, USA; 3 Internal Medicine, Interfaith Medical Center, Brooklyn, USA; 4 Preventive Medicine, Gerald Family Care, Washington, USA

**Keywords:** opioid use, prevalence, trend, depression, public health

## Abstract

Background

Depression and prescription opioid use have a bi-directional relationship. Depression commonly co-occurs with chronic noncancer pain and is known to be associated with opioid use. Studies have found an increased risk of depression only in patients with opioid dependence. Other studies have found an increased risk of opioid misuse in depressed patients. In addition, chronic pain conditions can lead to depression without the use of opioids.

Methods

We used the National Health and Nutrition Examination Survey (NHANES) data collected over seven survey cycles spanning 14 years: 2005/2006-2017/2018. Included in our study were participants ≥18 years who completed the patient health (PHQ-9) questionnaire. Persons with documented use of opioids were considered to have chronic use of opioids. Relevant data files were merged, and analytical weights computed in keeping with the survey analytical guidelines. Prevalence measures are reported as proportions. Associations were assessed using the Chi-square test. Binary logistic regression was used to assess the trend in the prevalence of opioid use. We used STATA-16 for data analysis and p-values <0.05 were considered statistically significant.

Results

A total of 36,459 participants met the inclusion criteria. The prevalence of depression was 7.7% (95% CI: 7.3-8.2). The prevalence of any narcotic use was 6.0%. Among depressed individuals, Blacks: OR 0.71 (95% CI: 0.54-0.93) and Hispanics: OR 0.48 (95% CI: 0.34-0.67) were less likely to be on narcotics compared to non-Hispanic Whites. The prevalence of opioid use was stable over the first 12 years, followed by a significant drop in the last two years.

Conclusion

Beyond the risk for opioid misuse, and opioid use disorder, depression should also be considered when prescribing opioids. It is therefore important to implement a training to screen for depression in patients receiving opioids for pain management.

## Introduction

In 1800s, the medicinal properties of opiates were recognized and were used by physicians to effectively alleviate suffering in patients. They were also used to treat cough, diarrhea, anxiety and minor pains [1,2]. Over the years, opioid use in the united states has been a public health crisis which still continues to escalate [3-6]. In the past two decades, the prescription of opioid analgesic has increased [7-9]. In 2012,259 million adults living in the United States had a prescription written order for opioid pain reliever [10]. This has posed a significant burden on the US health care system as the rate of opioid use disorders and opioid-related deaths have increased due to its increased use [11-13]. Opioid-related hospitalization increased between 2005 and 2014 accounting for 225 hospitalizations per 100,000 populations [3]. Death rate from opioid overdose increased to 27% from 2015 to 2016 [6].

Depression commonly co-occurs with chronic noncancer pain and is known to be associated with opioid use [14]. Some studies have found an increased risk of depression only in patients with opioid dependence [14]. Others have shown an increased risk of opioid misuse in patients with depression [15]. Also, chronic pain conditions can cause depression independent of opioid use [15]. Depression and prescription opioid use have a bi-directional relationship [15-17]. Depression is a well-established risk factor for receiving a prescription opioid for non-cancer pain, receiving opioids at higher doses and for longer duration, as well as elevating risk for both opioid misuse and opioid use disorder [14,16-21]. 

The main objective of this study is to explore the relationship between opioid use and depression, while describing the prevalence of opioid use in people with depression in the United States. The secondary objective is to measure the trend of opioid use in patients with depression.

## Materials and methods

Survey design

The National Health and Nutrition Examination Survey (NHANES) conducted by the National Center for Health Statistics (NCHS), is a nationally representative survey on the health and nutrition status of non-institutionalized US population. The survey utilizes a multistage probability sampling design to collect information from approximately 5,000 persons in 15 counties yearly. Details of the survey design and data collection have been previously published [22]. We utilized data collected over seven survey cycles spanning 14 years: 2005/2006-2017/2018. Ethical clearance was approved by the NCHS Research Ethics Review Board [22].

Data collection

Participants were interviewed at their homes to ascertain demographic characteristics. The interviewers were followed by the administration of the patient health questionnaire (PHQ-9) at mobile examination centers (MEC) to eligible participants. Included in our study were participants ≥18 years who completed the PHQ-9 questionnaire. Because of the sensitive nature of the questions, PHQ-9 questionnaires were not administered to participants needing proxies for completion. The PHQ-9 has been validated as an accurate screening tool for depression with scores ≥10 having a sensitivity and specificity of 88% across different study populations for the diagnosis of depression [22]. Prescription medication use is assessed by interviewers with visual confirmation using prescription packages. List of all medications considered as narcotics are listed in the appendix. Persons with documented use of opioids were considered to have chronic use of opioids. Persons who reported having smoked ≥100 cigarettes in their lifetimes were classified as smokers. The family poverty index ratio (PIR) was computed by dividing the total family income by the poverty threshold income, adjusting for family size. All study questionnaires, response options, and complete data, are publicly available.

Statistical analysis

Relevant data files were merged, and analytical weights computed following the survey analytical guidelines. Included in our analysis were participants ≥18 years with completed PHQ-9 questionnaires. Prevalence measures are reported as proportions. Associations were assessed using the Chi-square test. Binary logistic regression was used to assess the trend in prevalence of opioid use and graphically represented using a local polynomial smoothed line with 95% CI. We used STATA-16 for data analysis and p-values <0.05 were considered statistically significant.

## Results

In all, 36,459 met the inclusion criteria. The prevalence of depression in our study was 7.7% (95% CI: 7.3-8.2). Blacks: OR 1.3 (95% CI: 1.2-1.5) and Hispanics: OR 1.2 (95% CI 1.1-1.4) were more likely to be depressed compared to non-Hispanic Whites. The prevalence of any narcotic use was 6.0% in the general population and 17.2% in those with depression (see Table 1). Hispanics were less likely to use narcotics compared to non-Hispanic Whites and non-Hispanic Blacks. Among depressed individuals, Blacks: OR 0.71 (95% CI: 0.54-0.93) and Hispanics: OR 0.48 (95% CI: 0.34-0.67) were less likely to be on narcotics compared to non-Hispanic Whites.

**Table 1:** General characteristics of the study population. NH: non-Hispanic.

Variable	Categories	General population	Depressed	Not depressed
Age (years)		46.5	46.2	46.6
Body mass index (kg/m^2^)		29.0	30.6	28.8
Gender (%)	Male	48.8	36.2	49.9
	Female	51.2	63.8	50.1
Ethnicity (%)	NH White	67.5	63.1	67.8
	Hispanic	14.1	16.1	13.9
	NH Black	11.2	13.6	11.0
	Others	7.3	7.2	7.3
Educational status (%)	< High school	5.1	8.6	4.9
	High school or GED	10.5	17.0	9.9
	College	84.4	74.4	85.2
Marital status (%)	Married	63.3	47.6	64.6
	Divorced	18.5	31.1	17.4
	Never married	18.2	21.2	18.0
Country of birth (%)	USA	83.5	86.2	83.3
	Non-USA	16.5	13.8	16.7
Insurance (%)	Yes	82.5	76.3	83.0
Current smoker (%)	Yes	19.7	38.6	18.1
Ever smoker (%)	Yes	43.9	60.2	42.5
Poverty index ratio (%)	<1	13.4	27.6	12.2
	1-3	33.4	41.0	32.7
	>3	53.3	31.4	55.1
Narcotics use (%)	Yes	6.0	17.2	5.0
	No	94.0	82.8	95.0

The prevalence of opioid use was stable over the first 12 years, followed by a significant drop in the last two years (see Table 2).

**Table 2:** Trends in prevalence of opioid use among patients with depression.

Period	Prevalence of opioid use	P_delta_	P_trend_
2005-2008 (N=835)	18.5 (14.8-25.2)	-	0.069
2009-2012 (N=968)	17.8 (14.2-22.2)	0.812	
2013-2016 (N=940)	19.1 (14.5-24.9)	0.848	
2017-2018 (N=461)	10.7 (7.2-15.6)	0.015	

## Discussion

We looked at the prevalence of opioid use in patients with depression, using NHANES survey data. Responders with depression were more than three times more likely to be using opioids than those who were not depressed. Our study contributes to the growing literature on opioid use and risk of depression. It emphasizes that beyond the risk for opioid misuse, and opioid use disorder, depression should also be considered when prescribing opioids.

There appears to be a link between opioid use and depression, but the direction is uncertain given the cross-sectional nature of the NHANES survey. However, our findings are in keeping with other literature, suggesting that there is risk of new-onset or worsening depression with opioid use [14,15-21]. This increased risk is seen in both males and females [18].

There is an evidence to indicate that endogenous opioids have important roles in human mood and that endogenous mu and kappa opioid tone is dysregulated in the context of depression [23,24]. The underlying mechanisms of prescription opioids in the pathophysiology of depression are still unknown, but they may include opioid-induced dysregulation of reward circuitry, which results in decreased reward perception or pleasure and relief generation, or other physical medical dysregulation, which may contribute to the physical symptoms of depression. Studies have reported that prolonged opioid use (more than 30 days) contributes significantly to developing depression, even more than high doses of opioids [25-27].

Other studies have reported that the severity of depression is associated with increasing likelihood of misusing opioid medications for non-pain symptoms. Also, about one-third of patients who use long-term opioids and who have co-occurring depression, meet criteria for opioid use disorder. More than half of individuals with opioid use disorder have comorbid depression. This suggests that targeting opioid use prevention for patients with depression may help mitigate the US opioid epidemic [25].

There are some limitations to this study. The NHANES data is cross-sectional in nature, limiting its use for cause-effect analysis. Other limitations are related to the study design (survey), including recall bias, low response rate, and the fact that survey responders may not have provided accurate and honest answers to the questions. However, a strength of our study is that it represents the first to provide nationwide estimates of the prevalence of opioid use among patients with depression and using in person drug verifications to determine persons on narcotics allows for accurate determination of its use.

## Conclusions

Our study found that among people who reported taking opioids, the number of people who were depressed was more than three times those who were not depressed. It is therefore important to implement a training to screen for depression in patients receiving opioids for pain management.
